# Trends and forecasting of reported hepatitis B and hepatitis C incidence in Hainan Province, China, 2005–2020

**DOI:** 10.3389/fepid.2026.1899759

**Published:** 2026-08-31

**Authors:** Jiao Wang, Shibing Li, Cexiong Fu, Yi Su, Hanrong Fu, Yuanyuan Tian, Cheng Lan

**Affiliations:** 1 Department of Infectious Diseases, Hainan Afﬁliated Hospital of Hainan Medical University, Hainan General Hospital, Haikou, China; 2 Department of Pediatric Surgery, Hainan Afﬁliated Hospital of Hainan Medical University, Hainan General Hospital, Haikou, China; 3 Department of Hepatobiliary and Pancreatic Surgery, Hainan Afﬁliated Hospital of Hainan Medical University, Hainan General Hospital, Haikou, China; 4 Department of Gastroenterology, Hainan Afﬁliated Hospital of Hainan Medical University, Hainan General Hospital, Haikou, China

**Keywords:** Hainan Province, hepatitis B, hepatitis C, reported incidence, time-series analysis

## Abstract

**Objective:**

To assess long-term trends, seasonality, forecasts, and ecological associations with tourist visits for reported hepatitis B virus (HBV) and hepatitis C virus (HCV) incidence in Hainan Province from 2005 to 2020.

**Methods:**

Reported HBV and HCV incidence data from Hainan, national Global Burden of Disease (GBD) estimates for China, and annual tourist visit data were analyzed. Joinpoint regression assessed temporal trends, seasonal-trend decomposition using loess evaluated monthly variation, and seasonal autoregressive integrated moving average models generated forecasts. Spearman rank correlation analysis examined tourism associations, with sensitivity analyses excluding 2020 and using first-differenced and linearly detrended series.

**Results:**

Reported HBV incidence declined from 2005 to 2008, increased from 2008 to 2018, and then showed a non-significant decline through 2020; the overall average annual percent change (AAPC) was +3.37%. Reported HCV incidence increased until 2015 and subsequently declined, with an overall AAPC of +14.54%. National GBD estimates showed overall AAPCs of −0.54% for HBV and −1.52% for HCV. No significant differences among calendar months were detected. Under continuation of historical reporting patterns, the annualized projected reported HBV incidence in 2030 was 12.00 per 100,000 population (95% prediction interval: approximately 0.00–121.20), numerically close to the study-defined reference value of 12.20. The corresponding HCV estimate was 22.54 per 100,000 population (approximately 0.00–39.10), approximately 6.4 times the study-defined reference value of 3.55. The wide prediction intervals indicated substantial long-term forecast uncertainty. In 2020, observed mean monthly reported HBV and HCV incidence rates were 27.3% and 9.5% lower than forecasted values, respectively. In raw annual analyses, tourist visits correlated with reported HBV and HCV incidence *(r* = 0.871 and 0.879; both *P* < 0.001). After first differencing and linear detrending, the association remained significant only for reported HBV incidence.

**Conclusion:**

Reported HBV and HCV incidence in Hainan showed distinct phased changes without significant monthly differences. The forecasts represent projections under historical reporting patterns and should not be interpreted as evidence of progress toward WHO elimination targets. Tourism associations were exploratory and ecological and do not support causal inference. Continued surveillance and strengthened HCV screening, diagnosis, and linkage to treatment remain important.

## 1 Introduction

Viral hepatitis remains a major global public health concern. Hepatitis B virus (HBV) and hepatitis C virus (HCV) infections are important causes of chronic hepatitis, liver cirrhosis, and hepatocellular carcinoma, and they continue to impose substantial disease burdens across different regions worldwide, highlighting the long-term and complex nature of prevention and control efforts (1–3). Despite ongoing advances in vaccination, blood safety, antiviral therapy, and screening strategies, the large reservoir of chronic HBV infection, the high proportion of occult or undiagnosed HCV infection, and regional disparities in prevention and control capacity continue to hinder progress toward elimination targets (4, 5).

China is among the countries with a substantial burden of viral hepatitis. Previous studies have shown marked heterogeneity in HBV and HCV incidence trends across time, geographic areas, and population structures (6). In recent years, the management of HCV has changed substantially with the introduction of direct-acting antivirals (DAAs); however, insufficient screening coverage, delayed diagnosis, and disparities in access to treatment remain major barriers to HCV elimination (7). In addition, the COVID-19 pandemic may have affected healthcare-seeking behavior, testing, and disease reporting, leading to short-term fluctuations in viral hepatitis surveillance data (8, 9). Therefore, a regional-level integrated analysis of long-term trends, seasonal patterns, pandemic-related fluctuations, and future trajectories may help identify weaknesses in prevention and control.

Hainan Province is China's only tropical island province. It has a distinctive geographic and ecological environment and is also an important international tourism destination and free trade port, characterized by frequent population mobility and external exchanges. These features make its infectious disease surveillance and prevention context different from that of many inland regions (10–12). A previous study on HBV forecasting in Hainan also suggested that increased population mobility in the context of free trade port development may pose new challenges for the prevention and control of hepatitis B and other infectious diseases (13). Existing studies have highlighted the importance of travel-related infectious disease surveillance and HBV time-series forecasting in Hainan (13, 14). However, few studies have jointly examined reported HBV and HCV incidence in Hainan using long-term trend analysis, monthly decomposition, forecasting, pandemic sensitivity analysis, and ecological assessment of tourist visits.

Using reported HBV and HCV data from Hainan Province from 2005 to 2020, this study assessed long-term trends, monthly variation, projected reported incidence under historical patterns, and ecological associations with tourist visits. National Global Burden of Disease estimates were included as contextual references, and sensitivity analyses were conducted to assess the influence of 2020 and shared temporal trends. Study-defined 2030 reference values were used only for numerical comparison and were not considered equivalent to WHO elimination targets. The findings may support improvements in viral hepatitis surveillance, screening strategies, and regional resource allocation in Hainan and other settings with similar population-mobility patterns.

## 2 Methods

### 2.1 Study design and data sources

This retrospective ecological time-series study examined long-term trends, monthly variation, seasonality, forecasts, and ecological associations of reported hepatitis B and hepatitis C incidence in Hainan Province from 2005 to 2020. Annual and monthly reported case data were obtained from the Public Health Science Data Center (https://www.phsciencedata.cn/Share/) and accessed in January 2024. The HBV and HCV monthly series each contained 192 consecutive observations from January 2005 to December 2020. Population data were obtained from the Hainan Provincial Bureau of Statistics (https://stats.hainan.gov.cn) and accessed in April 2025. The year-end permanent resident population was used as the denominator, with the corresponding annual population used to calculate monthly reported incidence. National GBD-estimated HBV and HCV incidence rates for China from 1990 to 2023 were obtained from the Global Burden of Disease Results Tool (https://vizhub.healthdata.org/gbd-results/) and accessed in May 2025. Annual tourist visit data for Hainan Province from 2005 to 2020 were obtained from the Department of Tourism, Culture, Radio, Television and Sports of Hainan Province (https://lwt.hainan.gov.cn) and accessed in April 2025. Tourism data were matched by year with the corresponding annual reported HBV and HCV incidence rates. No missing or duplicate observations were identified, and no observations or study years were excluded. All data were aggregated, publicly available, and contained no personally identifiable information. The analyses used the versions of the datasets available on the stated access dates.

### 2.2 Incidence calculation and indicator definitions

The reported incidence rates of hepatitis B and hepatitis C were calculated as the number of reported cases divided by the corresponding permanent resident population and multiplied by 100,000, expressed as cases per 100,000 population. Annual reported incidence was calculated using annual reported cases and the corresponding annual population, whereas monthly reported incidence was calculated using monthly reported cases and the population of the corresponding year. The seasonal index (SI) was defined as the percentage of the average incidence level for each month relative to the overall monthly average. An SI of 100 represented the overall monthly average; SI > 100 indicated that the incidence level in a given month was higher than the overall monthly average, whereas SI < 100 indicated a lower-than-average level.

### 2.3 Joinpoint regression analysis

Joinpoint regression models were used to assess long-term changes in the reported incidence rates of hepatitis B and hepatitis C in Hainan Province and in GBD-estimated national incidence rates. This method identifies trend inflection points in time series and estimates the annual percent change (APC) for each segment and the average annual percent change (AAPC) over the entire observation period (15, 16). The analysis period was 2005–2020 for Hainan Province and 1990–2023 for national GBD-estimated incidence rates. Permutation tests were used to determine the number of joinpoints. Considering the number of annual observations, the maximum number of joinpoints was set to two for the Hainan reported incidence series and five for the national GBD-estimated incidence series. Hainan reported data and national GBD estimates were modeled separately. Because of differences in data sources and indicator definitions, national GBD-estimated trends were used only as contextual references.

### 2.4 STL decomposition and analysis of monthly variation patterns

Seasonal-trend decomposition using Loess (STL) was applied to the monthly reported incidence rates of HBV and HCV in Hainan Province from 2005 to 2020. An additive STL model was used to decompose each monthly time series into trend, seasonal, and remainder components (17), with a seasonal period of 12 months. The Kruskal–Wallis test was used to compare the distributions of the STL seasonal components across calendar months. Monthly SIs were additionally calculated to describe the magnitude of monthly variation relative to the overall monthly average. The SI was used for descriptive purposes and was not considered evidence of statistically significant seasonality.

### 2.5 SARIMA model development and forecasting

Seasonal autoregressive integrated moving average (SARIMA) models were developed using monthly reported hepatitis B and hepatitis C incidence rates in Hainan Province from 2005 to 2020. The models were specified as SARIMA (p,d,q) × (P,D,Q)_12_, where 12 represents the annual seasonal cycle of monthly data. Before model fitting, stationarity was assessed using differencing procedures, and autocorrelation function (ACF) and partial autocorrelation function (PACF) plots of the differenced series were examined to guide model specification. Candidate models with different combinations of non-seasonal and seasonal autoregressive and moving-average parameters were evaluated. Candidate models were first required to demonstrate satisfactory residual diagnostics, including no significant remaining residual autocorrelation. Among models with acceptable residual diagnostics, final model selection considered the Akaike information criterion (AIC), Bayesian information criterion (BIC), model parsimony, and supplementary forecast-error measures. The selected models were used to forecast monthly reported incidence rates from 2021 to 2030, with 95% prediction intervals. Annualized point forecasts were calculated as the sum of the 12 monthly point forecasts within each calendar year and were compared with the study-defined 2030 reference values, defined as 10% of the corresponding annual reported incidence rates in 2015.

### 2.6 Trend-based comparison with study-defined 2030 reference values

Referring to the WHO goal of a 90% reduction in new viral hepatitis infections by 2030 compared with 2015 (18), this study used the 2015 annual reported incidence rates of hepatitis B and hepatitis C in Hainan Province as the baseline and defined 10% of these values as the study-defined 2030 reference values. Because the analysis was based on statutory reported incidence rather than true new infection incidence, these reference values were used only for trend-based comparison and relative gap assessment. They are not equivalent to the WHO elimination targets and cannot be used to determine whether elimination has been or will be achieved. The gap ratio was defined as the ratio of the projected annualized reported incidence to the corresponding 2030 reference value. A ratio >1 indicated that the projected value remained above the reference level, whereas a ratio close to 1 indicated numerical proximity to the reference value under the continuation of historical reporting patterns.

### 2.7 Sensitivity analysis excluding 2020

To evaluate the influence of abnormal fluctuations in 2020 on trend estimation and forecasting, we conducted a Joinpoint exclusion sensitivity analysis and a SARIMA out-of-sample forecasting comparison. Joinpoint models were fitted using annual reported incidence rates for 2005–2020 and 2005–2019, respectively, and the corresponding AAPCs were compared. For SARIMA out-of-sample forecasting, models were developed using monthly reported incidence data from 2005 to 2019 to generate expected monthly reported incidence values for 2020 under continuation of the pre-pandemic historical pattern, which were then compared with the observed values in 2020. Relative differences were calculated using the predicted values as the reference; negative values indicated that the observed reported incidence was lower than the model-predicted value.

### 2.8 Exploratory ecological associations between tourist visits and reported HBV/HCV incidence

Ecological correlation analyses were conducted to evaluate associations between annual tourist visits and annual reported HBV and HCV incidence rates in Hainan Province. Annual tourist visits from 2005 to 2020 were matched with reported incidence rates for the corresponding years. Dual-axis time-series plots and ecological scatter plots were used for visualization. Crude associations were assessed using Spearman rank correlation analysis. Because tourism volume and reported hepatitis incidence could share long-term temporal trends, sensitivity analyses were additionally conducted using first-differenced and linearly detrended annual series. First differences were calculated as the value in year t minus the value in year t−1. For the detrended analysis, each variable was regressed separately on calendar year, and Spearman correlations were calculated between the resulting residuals. An additional analysis excluding 2020 was conducted to assess the influence of the COVID-19 pandemic year. These analyses were ecological and exploratory and were not intended to establish individual-level or causal relationships.

### 2.9 Statistical analysis

Joinpoint regression analyses were conducted using the Joinpoint Regression Program, version 5.2.0. Other analyses were performed using Python 3.11 and R 4.4.3. STL decomposition and HBV SARIMA modelling were conducted using *statsmodels* version 0.14.6, with forecasts generated using get_*forecast ()*. The HCV SARIMA model was fitted and forecast using *Arima ()* and *forecast ()*, respectively, in the R *forecast* package, version 9.0.2. Annual prediction intervals were estimated from 10,000 simulated forecast paths. Residual autocorrelation was assessed using the Ljung–Box test at lag 12 for HBV and lag 24 for HCV. Spearman correlations, first differencing, and linear detrending were performed using SciPy version 1.15.0, with 95% confidence intervals estimated from 10,000 paired bootstrap samples. All tests were two-sided, with *P* < 0.05 considered statistically significant.

## 3 Results

### 3.1 HBV and HCV reported incidence rates in Hainan Province exhibited segmented temporal patterns that differed from national GBD estimates

**Figure 1:**
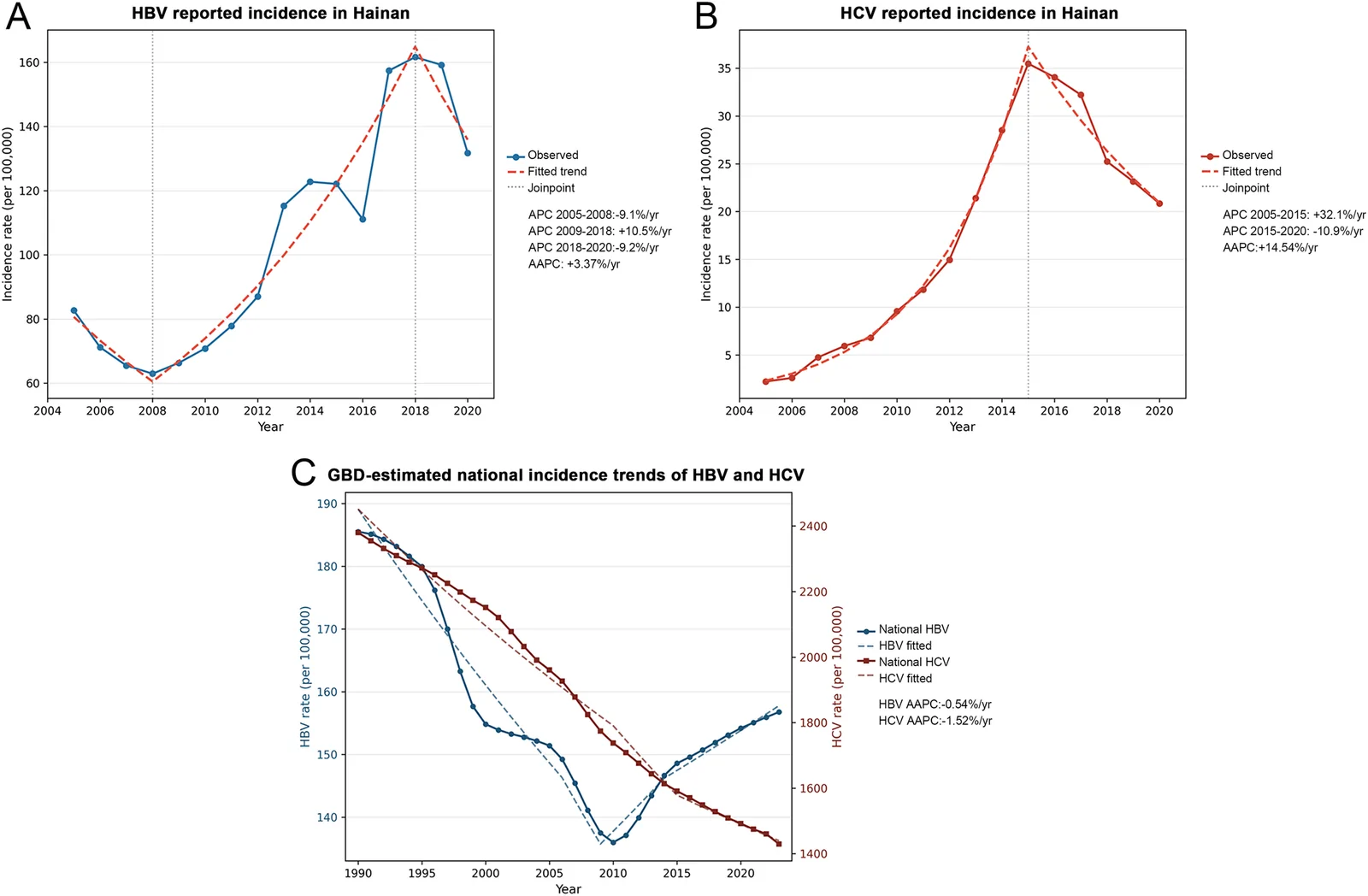
Joinpoint trend analysis of hepatitis B and hepatitis C incidence in Hainan Province and China. **(A)** Reported incidence trend of hepatitis B in Hainan Province from 2005 to 2020. **(B)** Reported incidence trend of hepatitis C in Hainan Province from 2005 to 2020. **(C)** GBD-estimated national incidence trends of hepatitis B and hepatitis C in China from 1990 to 2023. Dots and solid lines indicate observed incidence rates, dashed colored lines indicate Joinpoint-fitted trends, and vertical dotted lines indicate joinpoints. APC, annual percent change; AAPC, average annual percent change.

Joinpoint analysis showed that the reported incidence of hepatitis B in Hainan Province followed a phased pattern of decline, increase, and subsequent decline from 2005 to 2020. The incidence decreased from 2005 to 2008 (APC=−9.10%, *P* = 0.032), increased from 2008 to 2018 (APC=10.50%, *P* < 0.001), followed by an estimated decline from 2018 to 2020; however, this terminal segment was based on only three annual observations and was not statistically significant (APC=−9.20%, 95% CI: −52.03% to 71.86%; *P* = 0.305). The overall AAPC was 3.37% per year (*P* < 0.001) (Figure 1A, Table 1). The reported incidence of hepatitis C increased continuously from 2005 to 2015 (APC=32.10%, *P* < 0.001), followed by a decline from 2015 to 2020 (APC=−10.90%, *P* < 0.001). The overall AAPC was 14.54% per year (*P* < 0.001) (Figure 1B, Table 1). National GBD-estimated incidence rates showed that hepatitis B declined from 1990 to 2010 and increased thereafter, although the overall trend across the full observation period remained downward (AAPC=−0.54%, *P* < 0.001). Hepatitis C showed an overall downward trend from 1990 to 2023 (AAPC=1.52%, *P* < 0.001) (Figure 1C, Table 1).

**Table 1:** Joinpoint regression analysis of reported incidence rates in Hainan Province and GBD-estimated national incidence rates.

Data source	Disease	Period	Indicator	APC/AAPC (% per year)	95% CI (%)	*P* value
Hainan Province	HBV	2005–2008	APC	−9.10	(−15.68, −2.01)	0.032
Hainan Province	HBV	2008–2018	APC	10.5	(8.28, 12.76)	<0.001
Hainan Province	HBV	2018–2020	APC	−9.20	(−52.03, 71.86)	0.305
Hainan Province	HBV	2005–2020	AAPC	3.37	(1.44, 5.34)	<0.001
Hainan Province	HCV	2005–2015	APC	32.1	(29.47, 34.79)	<0.001
Hainan Province	HCV	2015–2020	APC	−10.90	(−14.14, −7.53)	<0.001
Hainan Province	HCV	2005–2020	AAPC	14.54	(12.89, 16.22)	<0.001
National estimates (GBD)	HBV	1990–2010	APC	−1.63	(−1.78, −1.47)	<0.001
National estimates (GBD)	HBV	2010–2015	APC	1.93	(1.59, 2.27)	<0.001
National estimates (GBD)	HBV	2015–2023	APC	0.69	(0.64, 0.73)	<0.001
National estimates (GBD)	HBV	1990–2023	AAPC	−0.54	(−1.45, −1.45)	<0.001
National estimates (GBD)	HCV	1990–2006	APC	−1.28	(−1.40, −1.17)	<0.001
National estimates (GBD)	HCV	2006–2009	APC	−2.72	(−2.92, −2.52)	<0.001
National estimates (GBD)	HCV	2009–2014	APC	−1.87	(−1.93, −1.81)	<0.001
National estimates (GBD)	HCV	2014–2023	APC	−1.28	(−1.34, −1.22)	<0.001
National estimates (GBD)	HCV	1990–2023	AAPC	−1.52	(−0.44%, −0.44)	<0.001

APC, annual percent change; AAPC, average annual percent change; CI, confidence interval.

### 3.2 STL decomposition and monthly variation patterns of the reported HBV incidence rate in Hainan Province

**Figure 2:**
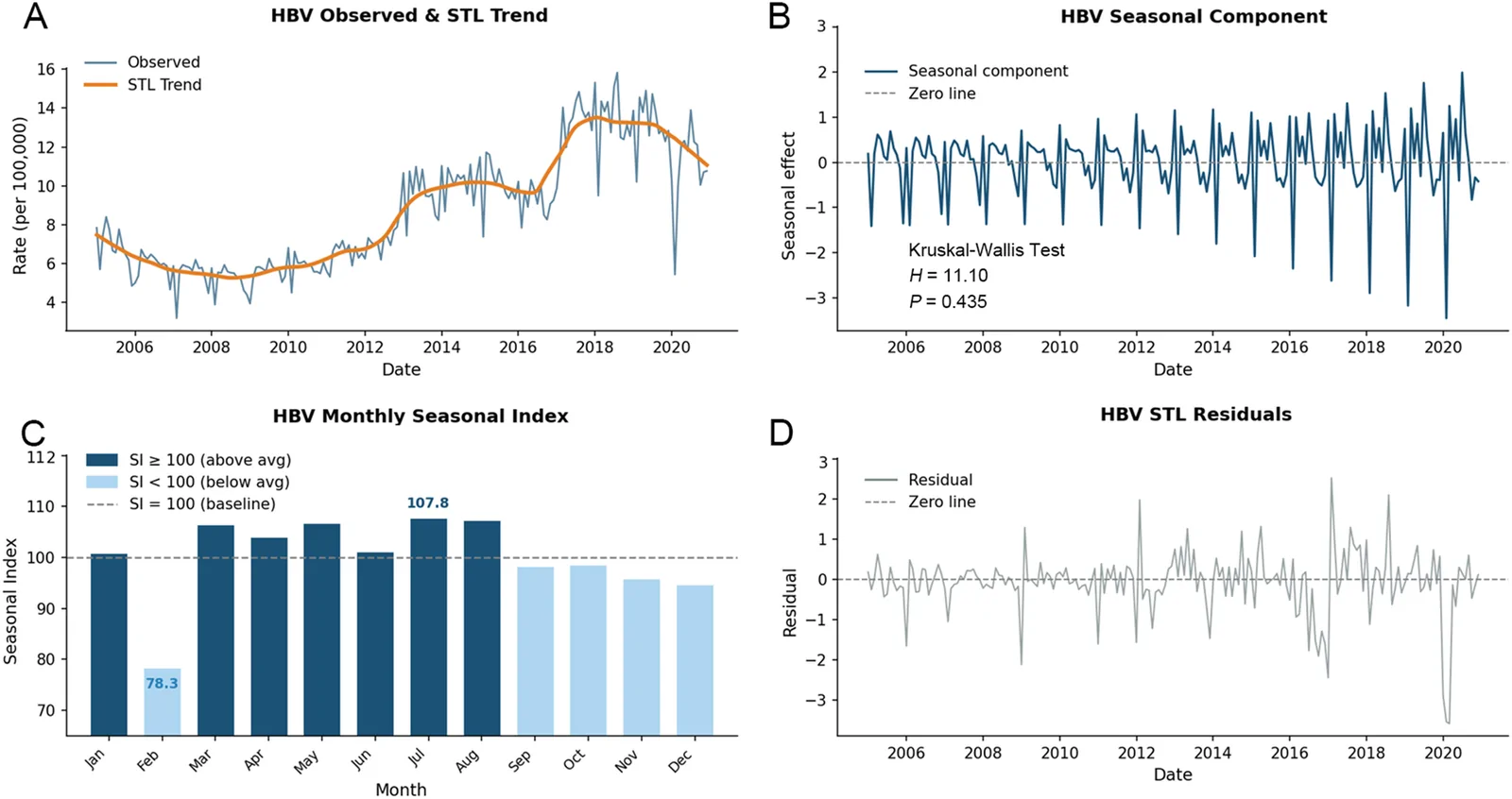
STL decomposition of monthly reported hepatitis B incidence in Hainan Province, 2005–2020. **(A)** Monthly observed values and STL trend component. **(B)** Seasonal component and Kruskal–Wallis test results. **(C)** Monthly seasonal indices, with the dashed line indicating the baseline value of 100. **(D)** STL residual component. Monthly incidence rates are expressed as cases per 100,000 population. SI, seasonal index; SI = 100 indicates the overall monthly average; KW, Kruskal–Wallis test.

From 2005 to 2020, the monthly reported HBV incidence rate in Hainan Province exhibited segmented temporal variation. The STL trend component declined from 2005 to 2008, subsequently increased gradually, rose more markedly after 2013, remained relatively high from 2017 to 2019, and declined in 2020 (Figure 2A). The seasonal component fluctuated around zero, with greater variation in later years than in earlier years. The Kruskal–Wallis test showed no statistically significant differences among calendar months (*H* = 11.10, *P* = 0.435; Figure 2B). Monthly SIs were generally above baseline from March to August and below baseline from September to December. The SI was highest in July (107.8) and lowest in February (78.3) (Figure 2C). The remainder component generally fluctuated around zero, with greater variation after 2016 and a marked negative deviation around 2020 (Figure 2D).

### 3.3 STL decomposition and monthly variation patterns of the reported HCV incidence rate in Hainan Province

**Figure 3:**
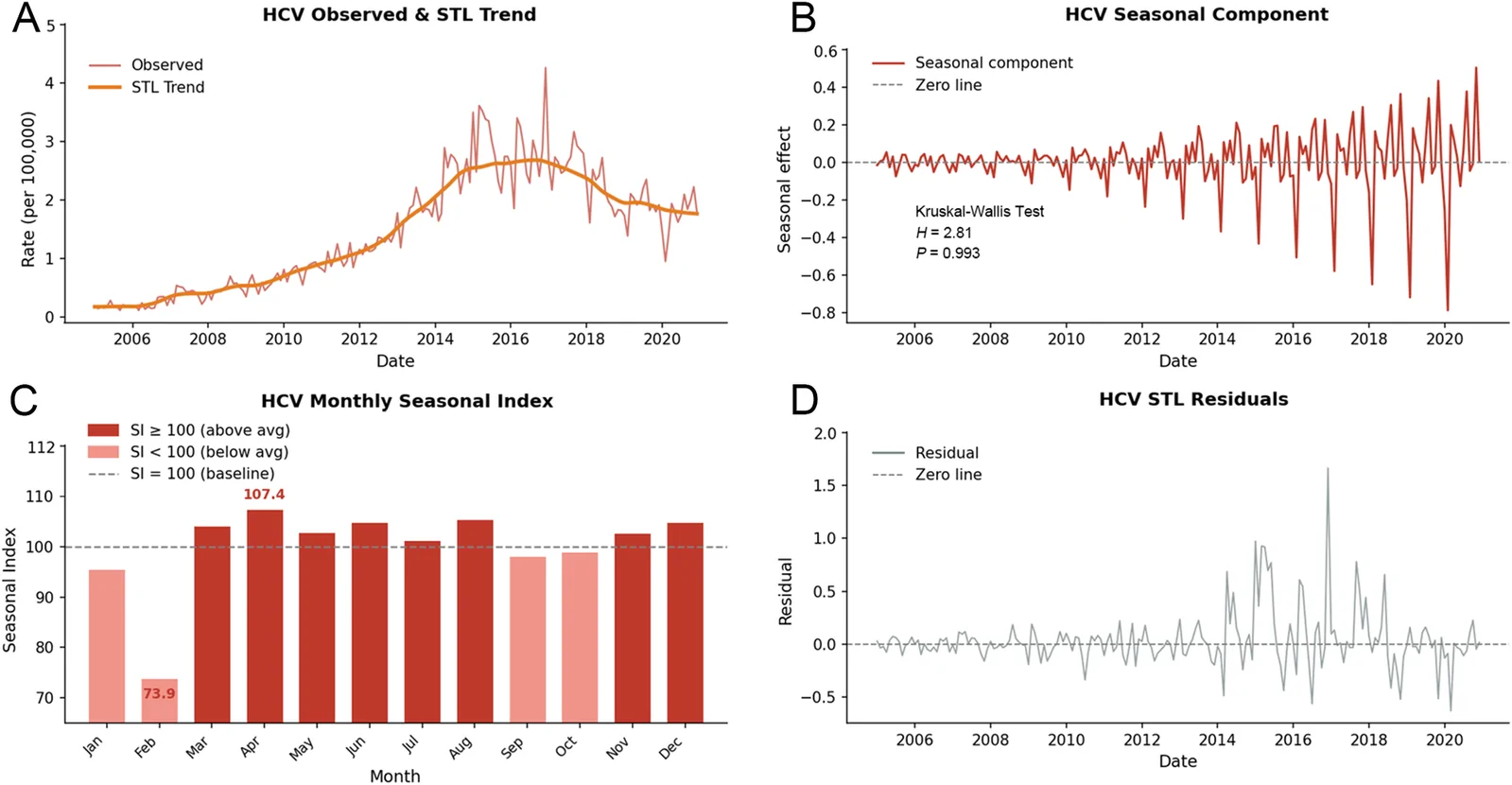
STL decomposition of monthly reported hepatitis C incidence in Hainan Province, 2005–2020. **(A)** Monthly observed values and STL trend component. **(B)** Seasonal component and Kruskal–Wallis test results. **(C)** Monthly seasonal indices, with the dashed line indicating the baseline value of 100. **(D)** STL residual component. Monthly incidence rates are expressed as cases per 100,000 population. SI, seasonal index; SI = 100 indicates the overall monthly average; KW, Kruskal–Wallis test.

From 2005 to 2020, the monthly reported HCV incidence rate in Hainan Province increased and subsequently declined. The STL trend component increased gradually from 2005 to 2013, remained relatively high from 2014 to 2016, and declined after 2017 (Figure 3A). The seasonal component fluctuated around zero, with greater variation after 2013 than in earlier years. The Kruskal–Wallis test showed no statistically significant differences among calendar months (*H* = 2.81, *P* = 0.993; Figure 3B). Monthly SIs were generally above baseline from March to August and from November to December and below baseline from January to February and from September to October. The SI was highest in April (107.4) and lowest in February (73.9) (Figure 3C). The remainder component generally fluctuated around zero, with relatively large variations around 2014–2017 and a marked positive deviation around 2017 (Figure 3D).

### 3.4 SARIMA forecasts of HBV and HCV reported incidence rates and comparisons with the 2030 reference values

**Figure 4:**
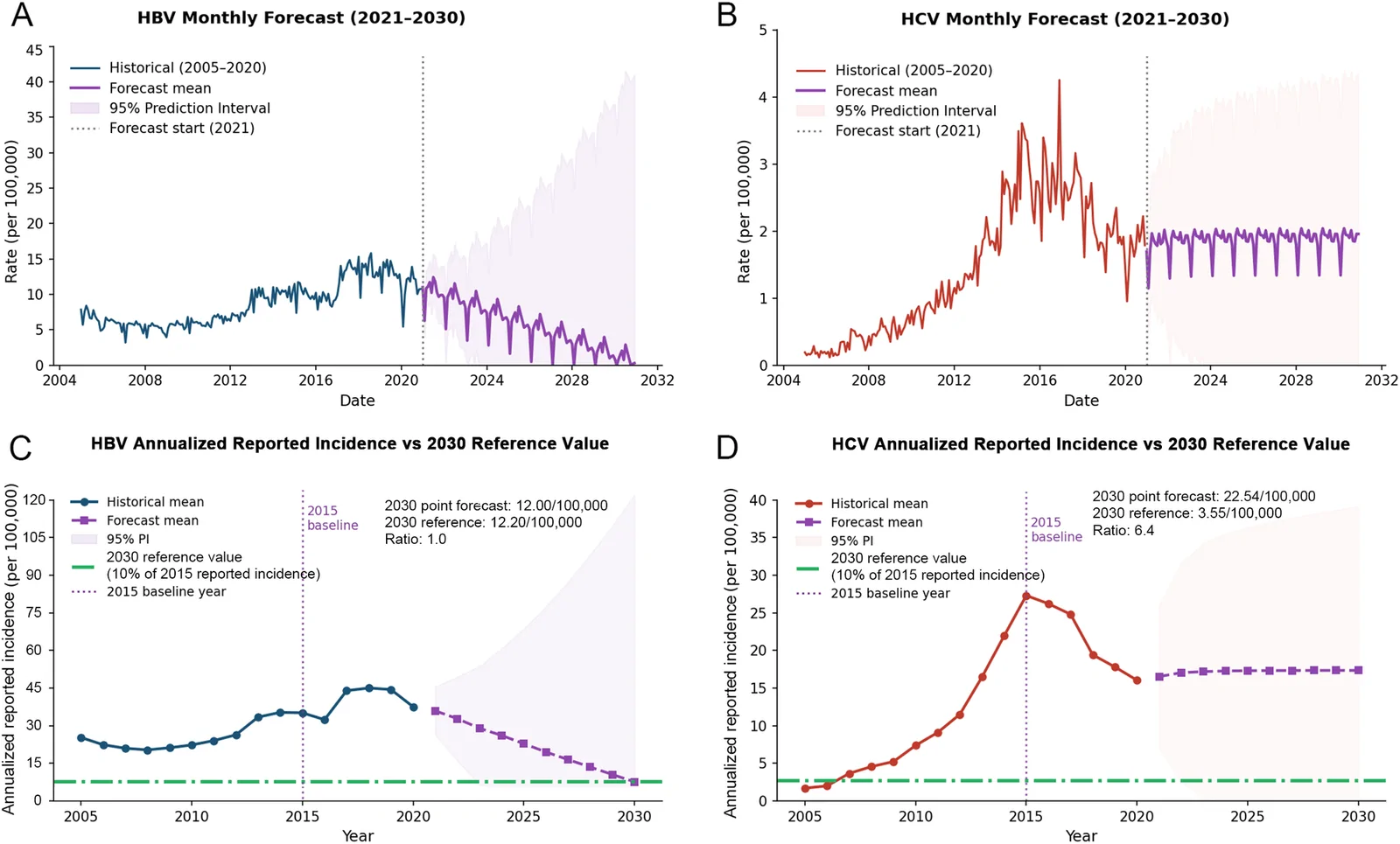
SARIMA forecasts of reported hepatitis B and hepatitis C incidence in Hainan Province and comparison with 2030 reference values. **(A)** SARIMA forecast of monthly reported hepatitis B incidence. **(B)** SARIMA forecast of monthly reported hepatitis C incidence. **(C)** Observed annual reported incidence, annualized forecasts, and 2030 reference value for hepatitis B. **(D)** Observed annual reported incidence, annualized forecasts, and 2030 reference value for hepatitis C. Shaded areas indicate 95% prediction intervals. The vertical dashed line indicates the start of the forecast period, and the horizontal dashed line indicates the corresponding 2030 reference value or one-fold reference level. The forecasts represent projections under the continuation of historical reporting patterns, and the reference values should not be interpreted as equivalent to WHO elimination targets.

Candidate SARIMA models were compared using AIC, BIC, residual diagnostics, and supplementary forecast-error measures (Supplementary Table 1). For HBV, SARIMA (0,1,2) × (2,1,0)_12_ was selected because it had the lowest AIC among the candidate models and showed no evidence of residual autocorrelation at lag 12 (Ljung–Box Q = 10.771, df = 12, *P* = 0.549). For HCV, SARIMA (1,1,1) × (0,0,1)_12_ was selected. Although an alternative model had slightly lower AIC and BIC values, its residuals retained significant autocorrelation. The selected HCV model showed acceptable residual diagnostics at lag 24 after adjustment for three fitted autoregressive and moving-average parameters (Ljung–Box Q = 31.162, df = 21, *P* = 0.074, Table 2). Prediction intervals widened substantially over the forecast horizon, indicating increasing uncertainty in the long-term projections. The annualized point forecast for reported HBV incidence declined to 12.00 per 100,000 population in 2030, with an approximate 95% prediction interval of 0.00–121.20per 100,000 population. The point forecast was numerically close to the study-defined 2030 reference value of 12.20 per 100,000 population derived from the 2015 baseline (Figure 4C). For HCV, the annualized point forecast changed only modestly and reached 22.54 per 100,000 population in 2030, with an approximate 95% prediction interval of 0.00–39.10 per 100,000 population. The HCV point forecast was approximately 6.4 times the corresponding study-defined reference value of 3.55 per 100,000 population (Figure 4D). The wide prediction intervals indicate that the numerical proximity of the HBV point forecast to its reference value, and the estimated HCV gap, should be interpreted cautiously. These projections represent conditional extrapolations of historical reported-incidence patterns and should not be interpreted as evidence of progress toward, or achievement of, WHO elimination targets.

**Table 2:** SARIMA model parameters and residual diagnostic results for monthly reported incidence rates of HBV and HCV in Hainan Province.

Disease	Final model	AIC	BIC	LB lag	Adjusted df	Ljung–Box Q	*P* value
HBV	SARIMA (0,1,2) × (2,1,0)_12_	471.56	486.77	12	12	10.771	0.549
HCV	SARIMA (1,1,1) × (0,0,1)_12_	77.95	90.96	24	21	31.162	0.074

SARIMA, seasonal autoregressive integrated moving average; AIC, Akaike information criterion; BIC, Bayesian information criterion; LB lag, maximum residual lag evaluated using the Ljung–Box test; adjusted df, effective degrees of freedom used for the test. For the HCV model, the adjusted degrees of freedom were calculated as the LB lag minus the number of fitted autoregressive and moving-average parameters. A *P* value <0.05 indicated statistically significant residual autocorrelation.

### 3.5 Sensitivity analyses excluding 2020

**Figure 5:**
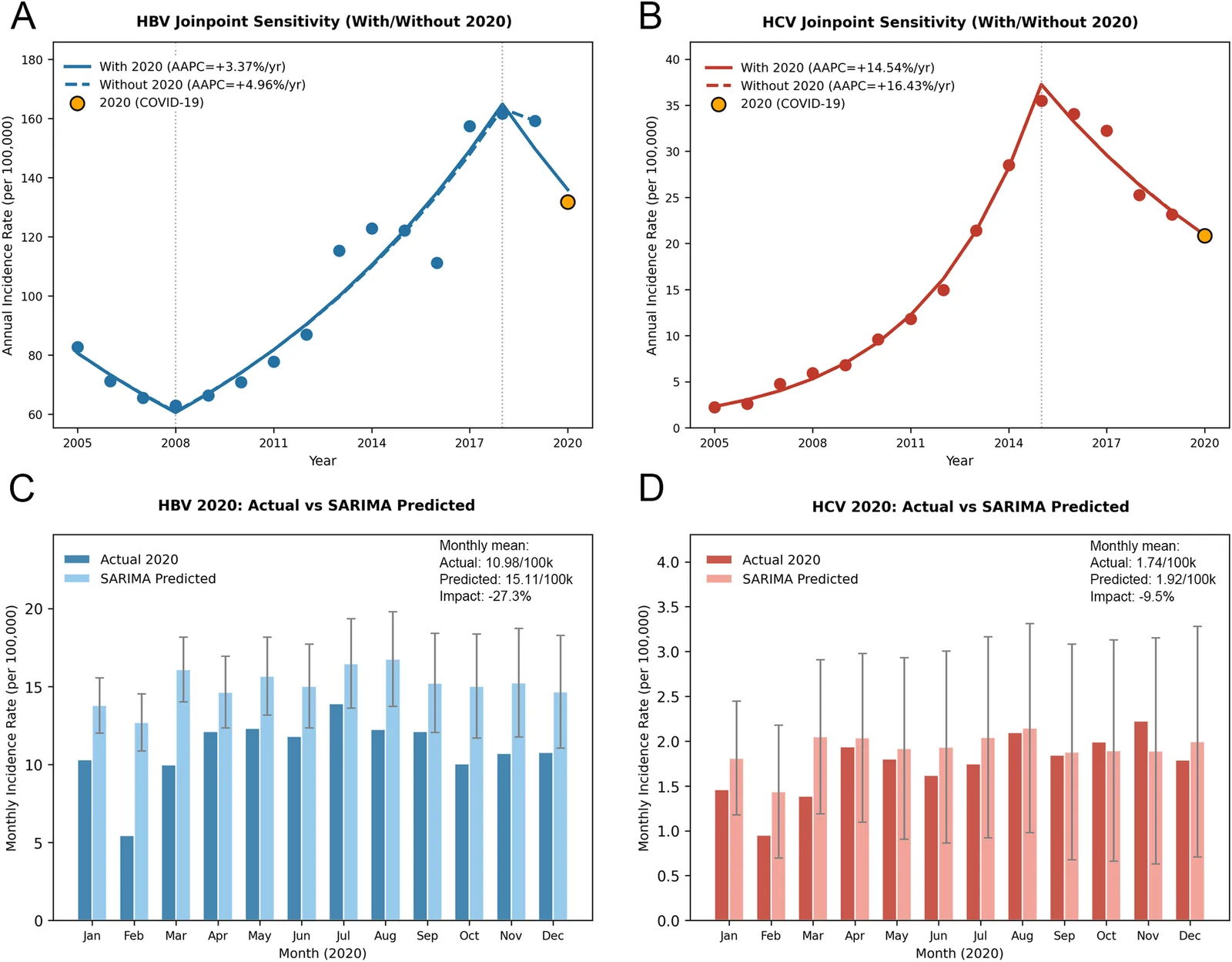
Sensitivity analysis for 2020 and SARIMA-predicted comparisons of reported hepatitis B and hepatitis C incidence in Hainan Province. **(A)** Comparison of Joinpoint trends in reported hepatitis B incidence with and without 2020. **(B)** Comparison of Joinpoint trends in reported hepatitis C incidence with and without 2020. **(C)** Observed monthly reported hepatitis B incidence in 2020 compared with SARIMA-predicted values. **(D)** Observed monthly reported hepatitis C incidence in 2020 compared with SARIMA-predicted values. Dots indicate annual observed values, orange dots indicate 2020 data, solid lines indicate fitted trends including 2020, and dashed lines indicate fitted trends excluding 2020.

In the HBV Joinpoint sensitivity analysis, the overall AAPC for the reported incidence rate in Hainan Province from 2005 to 2020 was 3.37% per year. After exclusion of 2020, the AAPC increased to 4.96% per year. The reported HBV incidence rate in 2020 was lower than those in 2018 and 2019 (Figure 5A). In the HCV Joinpoint sensitivity analysis, the overall AAPC from 2005 to 2020 was 14.54% per year. After exclusion of 2020, the AAPC increased to 16.43% per year. The reported HCV incidence rate in 2020 continued the declining trend observed after the 2015 peak (Figure 5B). In 2020, the observed mean monthly reported HBV incidence rate was 10.98 per 100,000 population, whereas the SARIMA forecast was 15.11 per 100,000 population. The observed value was 27.3% lower than the forecast value, and the observed monthly incidence rates were generally lower than the corresponding forecasts (Figure 5C). The observed mean monthly reported HCV incidence rate in 2020 was 1.74 per 100,000 population, whereas the SARIMA forecast was 1.92 per 100,000 population. The observed value was 9.5% lower than the forecast value, and the observed monthly incidence rates were generally lower than or close to the corresponding forecasts (Figure 5D).

### 3.6 Ecological correlations between annual tourist visits and HBV/HCV reported incidence rates in Hainan Province

**Figure 6:**
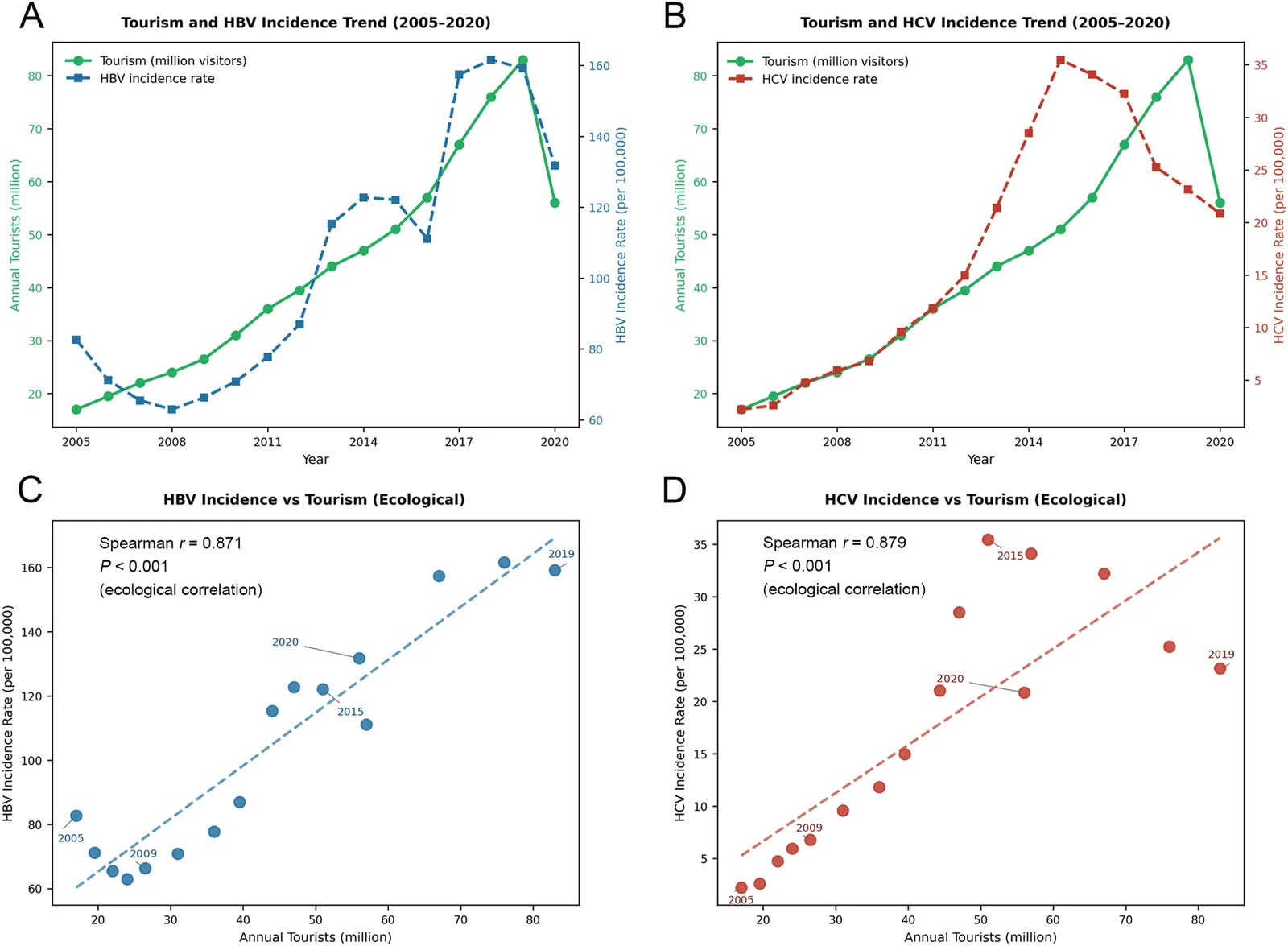
Annual changes and ecological correlations between tourist visits and reported hepatitis B/HCV incidence in Hainan Province, 2005–2020. **(A)** Annual trends in tourist visits and reported hepatitis B incidence in Hainan Province from 2005 to 2020. **(B)** Annual trends in tourist visits and reported hepatitis C incidence in Hainan Province from 2005 to 2020. **(C)** Scatter plot of reported hepatitis B incidence and annual tourist visits with fitted trend line. **(D)** Scatter plot of reported hepatitis C incidence and annual tourist visits with fitted trend line. The green line indicates annual tourist visits, and the blue and red lines indicate annual reported incidence rates of hepatitis B and hepatitis C, respectively. Point color indicates year. The fitted line represents the linear trend, and Spearman's r indicates the rank correlation coefficient. Results of first-difference, detrended, and 2020-exclusion sensitivity analyses are presented in Supplementary Table 2.

From 2005 to 2020, annual tourist visits to Hainan Province increased overall, from approximately 17 million visits in 2005 to approximately 83 million visits in 2019, before declining to approximately 56 million visits in 2020. During the same period, the reported HBV incidence rate declined from 2005 to 2008, subsequently increased overall, remained relatively high from 2017 to 2019, and declined in 2020 (Figure 6A). The reported HCV incidence rate increased continuously from 2005 to 2015, peaked in 2015, and subsequently declined (Figure 6B). Scatter-plot analyses showed that annual tourist visits were positively correlated with the reported HBV incidence rate (*r* = 0.871, *P* < 0.001; Figure 6C) and with the reported HCV incidence rate (*r* = 0.879, *P* < 0.001; Figure 6D). After excluding 2020, both associations remained significant (HBV: *r* = 0.857, *P* < 0.001; HCV: *r* = 0.896, *P* < 0.001). Because the annual series showed marked temporal trends, additional sensitivity analyses were performed using first-differenced and linearly detrended data. The association with reported HBV incidence remained significant after first differencing (*r* = 0.567, *P* = 0.028) and linear detrending (*r* = 0.691, *P* = 0.003). In contrast, the association with reported HCV incidence was no longer significant after first differencing (*r* = 0.070, *P* = 0.804) or linear detrending (*r* = 0.421, *P* = 0.105; Supplementary Table 2). These findings suggest that the HBV association was more robust, whereas the HCV association was largely influenced by shared temporal trends.

## 4 Discussion

This study showed that reported HBV and HCV incidence in Hainan Province from 2005 to 2020 exhibited clear phased changes and was not fully consistent with national GBD-estimated trends. At the national level, HBV incidence showed a long-term downward trend, which may be attributable to the inclusion of hepatitis B vaccination in the childhood immunization program, birth cohort effects, and sustained public health interventions. Studies of acute hepatitis B and hepatitis B in Guangzhou have also reported similar declining trends, suggesting that immunization strategies may have gradually conferred protection in younger birth cohorts (19, 20). In contrast, reported HBV incidence in Hainan increased over a relatively long period after 2008. This pattern may reflect changes in underlying disease occurrence as well as case-detection capacity, healthcare accessibility, screening activity, and reporting-system sensitivity. National spatial epidemiological studies have shown substantial geographic heterogeneity in HBV incidence in China, with population size, rural population proportion, and their interactions influencing reported levels. These findings provide contextual evidence for interpreting differences between Hainan and national trends (6, 21).

The reported HBV incidence trend in Hainan also differed from reported trends in Guangzhou, Xiamen, and other cities. In Guangzhou, both chronic and acute hepatitis B incidence decreased from 2008 to 2022, and in Xiamen, hepatitis B incidence generally declined from 2004 to 2022. However, these studies also indicated that adult infection burden and ongoing prevention and control pressures remain important (20, 22). In the present study, the mid-period increase and subsequent decline in reported HBV incidence in Hainan may reflect several overlapping processes. Expanded screening, increased detection of previously acquired chronic infections, repeated healthcare encounters, and changes in reporting practices may have contributed to the observed increase, whereas increasing vaccine-derived protection may have contributed to the later decline. Previous research has also indicated that China still has a large reservoir of chronic HBV infection, and insufficient diagnosis, treatment, and follow-up remain major barriers to achieving elimination goals (23). Therefore, the phased increase in reported HBV incidence in Hainan should not be interpreted simply as a failure of vaccination strategy. Rather, it may reflect the combined effects of immunization progress, increased detection of existing infections, population mobility, and reporting behavior.

For HCV, reported incidence in Hainan increased rapidly before 2015 and subsequently declined, showing partial consistency with national GBD-estimated trends and findings from some coastal regions. Studies on the HCV disease burden in China have shown that age-standardized HCV incidence has generally declined since 1990, although undiagnosed infection and aging-related burden remain persistent challenges (24, 25). A study from the southeastern coastal province of Fujian reported a decline in HCV incidence from 2015 to 2022, which is consistent with the post-2015 decline observed in Hainan (26). The increase in reported HCV incidence in Hainan before 2015 may have reflected improved testing capacity, strengthening of the reporting system, increased screening, or detection of previously undiagnosed infections. The decline in reported HCV incidence after 2015 coincided with broader improvements in blood safety, awareness, screening, and access to antiviral treatment. However, because Hainan-specific information on testing and treatment coverage was unavailable, the present analysis cannot determine whether these factors caused the observed decline. Although DAAs can reduce the prevalence of chronic HCV infection and the proportion of undiagnosed cases, a large pool of undiagnosed infections may continue to limit progress toward elimination (27, 28).

No statistically significant differences among calendar months were detected in reported HBV or HCV incidence; the observed fluctuations may reflect reporting variation rather than seasonal transmission. Previous HBV forecasting research in Hainan and national time-series studies of HBV and HCV have reported low values in February and relatively high values in some months (13, 29). In the present study, the relatively low seasonality index in February may be related to reduced healthcare seeking, delayed testing, and altered reporting behavior during the Spring Festival holiday. However, the Kruskal–Wallis test did not reach statistical significance, indicating that these differences were insufficient to constitute a stable seasonal pattern. Because HBV and HCV are often associated with chronic infection, screening-based detection, and medical reporting, long-term trends and population-structure changes may have greater public health relevance than isolated monthly peaks and troughs.

Under continuation of historical reporting patterns, the projected reported HBV incidence may approach the study-defined 2030 reference value, whereas projected reported HCV incidence may remain substantially above the corresponding reference value. These numerical comparisons should not be interpreted as evidence of progress toward, or achievement of, WHO elimination targets. These findings suggest that, under current trajectories, HBV and HCV prevention and control face different challenges. HBV control relies more heavily on long-term immunization coverage, prevention of mother-to-child transmission, adult diagnosis and treatment, and management of chronic infection. In contrast, HCV elimination depends more on active screening, rapid linkage to treatment after diagnosis, and improved treatment coverage (30, 31). Notably, although reported HCV incidence in Hainan declined after 2015, he projected point estimate remained above the study-defined 2030 reference value derived from the WHO 90% reduction framework, indicating that a declining trend does not necessarily imply proximity to elimination. DAAs alone are unlikely to automatically achieve HCV elimination; insufficient screening, incomplete linkage to care, declining treatment uptake, and ongoing transmission in high-risk populations may all weaken control efforts (32–34).

This study integrated monthly reported data from Hainan Province, Joinpoint segmented trend analysis, STL seasonal decomposition, SARIMA forecasting, and numerical comparison with study-defined 2030 reference values derived from the WHO 90% reduction framework within a unified analytical framework. It not only described historical changes in HBV and HCV but also assessed how model-based point forecasts compared numerically with the study-defined 2030 reference values. Previous studies have suggested that HBV and HCV still require intensified interventions to achieve 2030 goals, and local forecasting models may provide useful evidence for early warning and resource allocation (22, 29). Therefore, this study provides quantitative evidence for optimizing viral hepatitis prevention and control strategies and allocating resources more precisely in Hainan Province.

The 2020 sensitivity analysis suggested that the COVID-19 pandemic may have affected viral hepatitis surveillance data in Hainan. The observed reported incidence of HBV and HCV in 2020 was lower than expected from pre-pandemic historical patterns. The estimated AAPCs also increased after 2020 was excluded. Thus, the 2020 observations deviated from previous reporting patterns and influenced the estimated long-term trends. Reduced healthcare-seeking, testing, or reporting may partly explain these differences. Studies from China and other countries have also shown that pandemic-control measures, altered healthcare-seeking behavior, reduced screening, reporting delays, and disruptions in treatment services may affect viral hepatitis surveillance data (35, 36). Therefore, the decline in reported incidence in Hainan in 2020 should be interpreted as the result of combined influences from changes in true transmission, reduced healthcare accessibility, and disturbances in the reporting system, rather than as evidence of a sudden improvement in prevention and control.

This study found positive ecological correlations between tourist visits and reported HBV and HCV incidence in the unadjusted analyses. After first differencing and linear detrending, the association with reported HBV incidence remained statistically significant, whereas the association with reported HCV incidence was no longer significant. The latter was therefore likely influenced by shared temporal trends, while the HBV finding should still be interpreted cautiously. Tourism volume may reflect concurrent changes in population mobility, healthcare use, screening activity, socioeconomic development, and reporting capacity rather than a direct effect on hepatitis transmission. Studies among travelers and port populations have also highlighted the importance of mobility-related infectious disease surveillance (11, 37, 38). Therefore, the association between tourism volume and reported HBV/HCV incidence in Hainan may more likely reflect concurrent changes in mobile populations, medical testing, socioeconomic development, and reporting capacity, rather than a direct causal effect of tourism activity on transmission.

This study has several limitations. First, it was based on aggregated data and lacked individual-level information on age, sex, vaccination status, transmission route, testing, and treatment, which precluded identification of specific risk factors or causal inference. Second, reported incidence may be affected by surveillance capacity, healthcare-seeking behavior, and changes in reporting practices, and therefore may not fully represent the true infection level. Third, Hainan reported data and national GBD estimates were derived from different data sources and are suitable only for trend comparison. Fourth, the SARIMA forecasts did not incorporate external variables such as policy interventions, population mobility, or treatment coverage, resulting in forecasting uncertainty. Finally, the association between tourism volume and reported HBV/HCV incidence was ecological and cannot directly demonstrate that tourism mobility caused changes in incidence.

Future studies should integrate individual-level surveillance data and multisource data to further investigate the specific drivers of HBV and HCV incidence changes. Variables such as vaccination coverage, screening coverage, antiviral treatment, population mobility, and healthcare resources should be incorporated to develop more refined forecasting models. Given the tourism and population mobility characteristics of Hainan, future work should strengthen continuous surveillance among mobile populations and key populations, and evaluate the real-world effects of screening, diagnosis, and treatment interventions on progress toward independently defined hepatitis elimination indicators.

In conclusion, reported HBV and HCV incidence in Hainan Province showed distinct phased changes from 2005 to 2020, with no statistically significant differences among calendar months. Under continuation of historical reporting patterns, forecasts suggested that reported HBV incidence may approach the study-defined 2030 reference value, whereas reported HCV incidence may remain above it; these projections should not be interpreted as evidence of progress toward WHO elimination targets. The associations with tourist visits were exploratory and ecological, and neither supports causal inference. Hainan Province should continue to strengthen viral hepatitis surveillance, maintain HBV immunization and screening, and prioritize HCV screening, diagnosis, and linkage to treatment.

## Data Availability

Publicly available datasets were analyzed in this study. This data can be found here: The datasets analyzed in this study are publicly available from the following sources: Public Health Science Data Center of China: https://www.phsciencedata.cn/Share/Global Burden of Disease Results Tool:https://vizhub.healthdata.org/gbd-results/Hainan Provincial Bureau of Statistics: https://stats.hainan.gov.cn/Department of Tourism, Culture, Radio, Television and Sports of Hainan Province:https://lwt.hainan.gov.cn/No accession numbers are applicable.

## References

[1] Collaborators POCEahcoPO. Number of people treated for hepatitis C virus infection in 2014–2023 and applicable lessons for new HBV and HDV therapies. J Hepatol. (2025) 83:329–347. 10.1016/j.jhep.2025.01.013 39914746 PMC12278943

[2] Alberts CJ Clifford GM Georges D Negro F Lesi OA Hutin YJ-F. Worldwide prevalence of hepatitis B virus and hepatitis C virus among patients with cirrhosis at country, region, and global levels: a systematic review. Lancet Gastroenterol Hepatol. (2022) 7:724–735. 10.1016/s2468-1253(22)00050-4 35576953 PMC9259503

[3] Xiao J Wang F Yuan Y Gao J Xiao L Yan C. Epidemiology of liver diseases: global disease burden and forecasted research trends. Sci China Life Sci. (2025) 682:541–557. 10.1007/s11427-024-2722-2 39425834

[4] Cui F Blach S Manzengo Mingiedi C Gonzalez MA Sabry Alaama A Mozalevskis A. Global reporting of progress towards elimination of hepatitis B and hepatitis C. Lancet Gastroenterol Hepatol. (2023) 84:332–342. 10.1016/s2468-1253(22)00386-7 36764320

[5] Kaewdech A Charatcharoenwitthaya P Piratvisuth T. Asian Perspective on hepatitis B virus and hepatitis C virus elimination. Viruses. (2024) 171:34. 10.3390/v17010034 PMC11768638 39861823

[6] Qian J Yue M Huang P Ai L Zhu C Wang C. Spatiotemporal heterogeneity and impact factors of hepatitis B and C in China from 2010 to 2018: bayesian space-time hierarchy model. Front Cell Infect Microbiol. (2023) 13:1115087. 10.3389/fcimb.2023.1115087 36923590 PMC10008934

[7] Zhao Z Chu M Guo Y Yang S Abudurusuli G Frutos R. Feasibility of hepatitis C elimination in China: from epidemiology, natural history, and intervention perspectives. Front Microbiol. (2022) 13:884598. 10.3389/fmicb.2022.884598 35722351 PMC9201439

[8] He CQ Sun BH Yu WT An SY Qiao BJ Wu W. Evaluating the impact of COVID-19 outbreak on hepatitis B and forecasting the epidemiological trend in mainland China: a causal analysis. BMC Public Health. (2024) 241:47. 10.1186/s12889-023-17587-3 PMC10763123 38166922

[9] Ramos-Rincon JM Pinargote-Celorio H De Mendoza C Ramos-Belinchón C Moreno-Torres V Treviño A. Impact of the COVID-19 pandemic on hospital admissions due to viral hepatitis in Spain. J Clin Virol. (2023) 167:105553. 10.1016/j.jcv.2023.105553 37549555

[10] Zhang T Ren G Zhou H Qiang Y Li J Zhang Y. Molecular prevalence and genetic diversity analysis of enterocytozoon bieneusi in humans in Hainan Province, China: high diversity and unique endemic genetic characteristics. Front Public Health. (2022) 10:1007130. 10.3389/fpubh.2022.1007130 36148343 PMC9485493

[11] Chen J Yang J Huang S Li X Liu G. Forecasting tourist arrivals for hainan island in China with decomposed broad learning before the COVID-19 pandemic. Entropy (Basel). (2023) 252:338. 10.3390/e25020338 PMC9954797 36832704

[12] Qi F Wu Y Wang J Wang Q. China’s hainan free trade port: medical laws and policy reform. Front Public Health. (2021) 9:764977. 10.3389/fpubh.2021.764977 34869178 PMC8634373

[13] Fang K Cao L Fu Z Li W. Prediction of reported monthly incidence of hepatitis B in Hainan Province of China based on SARIMA-BPNN model. Medicine (Baltimore). (2023) 10241:e35054. 10.1097/md.0000000000035054 PMC10578744 37832091

[14] Luo Z Wang W Ding Y Xie J Lu J Xue W. Epidemiological characteristics of infectious diseases among travelers between China and foreign countries before and during the early stage of the COVID-19 pandemic. Front Public Health. (2021) 9:739828. 10.3389/fpubh.2021.739828 34869153 PMC8634889

[15] Kim HJ Fay MP Feuer EJ Midthune DN. Permutation tests for joinpoint regression with applications to cancer rates. Stat Med. (2000) 193:335–351. 10.1002/(sici)1097-0258(20000215)19:3<335::aid-sim336>3.0.co;2-z 10.1002/(sici)1097-0258(20000215)19:3<335::aid-sim336>3.0.co;2-z 10649300

[16] Clegg LX Hankey BF Tiwari R Feuer EJ Edwards BK. Estimating average annual per cent change in trend analysis. Stat Med. (2009) 2829:3670–3682. 10.1002/sim.3733 PMC2843083 19856324

[17] Ke G Hu Y Huang X Peng X Lei M Huang C. Epidemiological analysis of hemorrhagic fever with renal syndrome in China with the seasonal-trend decomposition method and the exponential smoothing model. Sci Rep. (2016) 6:39350. 10.1038/srep39350 27976704 PMC5157041

[18] Hutin YJ Bulterys M Hirnschall GO. How far are we from viral hepatitis elimination service coverage targets? J Int AIDS Soc. (2018) 21(Suppl 2):e25050. 10.1002/jia2.25050 29633520 PMC5978704

[19] Wang L Liu N Miao N Zhang G Wang F Wang H. Trends and age-period-cohort effect on acute hepatitis B incidence—china, 2005–2021. China CDC wkly. (2024) 630:727–733. 10.46234/ccdcw2024.164 PMC11301606 39114316

[20] Zheng Z Lin X Huang Y Zhang C Zhang Z. Trends and age-period-cohort effect on incidence of hepatitis B from 2008 to 2022 in Guangzhou, China. Sci Rep. (2024) 141:13370. 10.1038/s41598-024-63796-0 PMC11166960 38862511

[21] Fang K Cheng N Nie C Song W Zhao Y Pan J. Spatial and temporal distribution patterns and factors influencing hepatitis B in China: a geo-epidemiological study. BMC Public Health. (2025) 251:1276. 10.1186/s12889-025-22452-6 PMC11971818 40186148

[22] Zhang R Mi H He T Ren S Zhang R Xu L. Trends and multi-model prediction of hepatitis B incidence in Xiamen. Infect Dis Model. (2024) 94:1276–1288. 10.1016/j.idm.2024.08.001 PMC11366886 39224908

[23] Yan R Sun M Yang H Du S Sun L Mao Y. 2024 Latest report on hepatitis B virus epidemiology in China: current status, changing trajectory, and challenges. Hepatobiliary Surg Nutr. (2025) 141:66–77. 10.21037/hbsn-2024-754 PMC11806133 39925891

[24] Lin F Liu Z Zhai J Yi S Li Y Wang Y. Age-period-cohort and Bayesian age-period-cohort prediction modeling of hepatitis C incidence and mortality in China. BMC Public Health. (2025) 251:2079. 10.1186/s12889-025-22847-5 PMC12135535 40468268

[25] Yang J Qi J-L Wang X-X Li X-H Jin R Liu B-Y. The burden of hepatitis C virus in the world, China, India, and the United States from 1990 to 2019. Front Public Health. (2023) 11:1041201. 10.3389/fpubh.2023.1041201 36935711 PMC10018168

[26] Liu W Lian Q Li Z Lu X Wu S Zhang M. Epidemiological characteristics and spatiotemporal distribution of hepatitis C in southeast coastal areas of China from 2015 to 2022. BMC Infect Dis. (2025) 251:394. 10.1186/s12879-025-10778-w PMC11929232 40119257

[27] Forouzannia F Hamadeh A Passos-Castilho AM Erman A Yu A Feng Z. Impact of new direct-acting antiviral therapy on the prevalence and undiagnosed proportion of chronic hepatitis C infection. Liver Int. (2024) 446:1383–1395. 10.1111/liv.15875 38445848

[28] Yew KC Tan QR Lim PC Low WY Lee CY. Assessing the impact of direct-acting antivirals on hepatitis C complications: a systematic review and meta-analysis. Naunyn Schmiedebergs Arch Pharmacol. (2024) 3973:1421–1431. 10.1007/s00210-023-02716-x 37728622

[29] Wang Y Qing S Liang Z Ma C Bai Y Xu C. Time series analysis-based seasonal autoregressive fractionally integrated moving average to estimate hepatitis B and C epidemics in China. World J Gastroenterol. (2023) 2942:5716–5727. 10.3748/wjg.v29.i42.5716 PMC10701333 38075851

[30] Yao L Lin S Huang J Wu Y. Burden of hepatitis B-associated diseases in China from 1990 to 2030. Zhongguo Xue Xi Chong Bing Fang Zhi Za Zhi. (2023) 355:464–475. 10.16250/j.32.1374.2023068 38148535

[31] Zhou M Yao L Wu Y Lin S Huang J. Analysis and prediction of burden of viral hepatitis C-associated diseases in China from 1990 to 2044. Zhongguo Xue Xi Chong Bing Fang Zhi Za Zhi. (2023) 355:476–485. 10.16250/j.32.1374.2023059 38148536

[32] Kwon JA Dore GJ Hajarizadeh B Alavi M Valerio H Grebely J. Australia Could miss the WHO hepatitis C virus elimination targets due to declining treatment uptake and ongoing burden of advanced liver disease complications. PLoS One. (2021) 169:e0257369. 10.1371/journal.pone.0257369 PMC8445464 34529711

[33] Lazarus JV Picchio CA Byrne CJ Crespo J Colombo M Cooke GS. A global systematic review of hepatitis C elimination efforts through micro-elimination. Semin Liver Dis. (2022) 422:159–172. 10.1055/a-1777-6112 35189667

[34] Zhang S Cui F. Global progress, challenges and strategies in eliminating public threat of viral hepatitis. Infect Dis Poverty. (2025) 141:9. 10.1186/s40249-025-01275-y PMC11806526 39923069

[35] Liu J Zeng W Zhuo C Liu Y Zhu L Zou G. Impact of the COVID-19 pandemic on the incidence of notifiable infectious diseases in China based on SARIMA models between 2013 and 2021. J Epidemiol Glob Health. (2024) 14:273. 10.1007/s44197-024-00273-x PMC11442807 39080246

[36] Russotto A Vicentini C Ferrigno L Crateri S Russo R Tosti ME. Impact of the COVID-19 pandemic on the Italian national viral hepatitis surveillance: an interrupted time series analysis, 2006–2022. Public Health. (2024) 232:14–20. 10.1016/j.puhe.2024.04.006 38728904

[37] Ju Z Zhou X Li P Lu Y Qin J. Decade-Long analysis of hepatitis trends among international travelers at Shanghai port (2013–2023). Med Sci Monit. (2025) 31:e948512. 10.12659/msm.948512 40531804 PMC12184213

[38] Zhang D Ge Y Wang J Liu H Zhang W-B Wu X. Optimizing the detection of emerging infections using mobility-based spatial sampling. Int J Appl Earth Obs Geoinf. (2024) 131:103949. 10.1016/j.jag.2024.103949 38993519 PMC11234252

