# Supplementary material for: Trends and forecasting of reported hepatitis B and hepatitis C incidence in Hainan Province, China, 2005–2020
Source: Front Epidemiol. 2026 Aug 31;6:1899759. doi: 10.3389/fepid.2026.1899759 (PMC13572582; doi:10.3389/fepid.2026.1899759)
Supplement: Supplementary file 1 [file Table1.docx]

Supplementary Table S1. Candidate SARIMA models and diagnostic criteria for monthly reported HBV and HCV incidence in Hainan Province, 2005–2020

| Disease | Model | SARIMA specification | AIC | BIC | LB lag | fitdf | Test df | Ljung–Box Q | *P* value | MAPE (%) | RMSE |
| --- | --- | --- | --- | --- | --- | --- | --- | --- | --- | --- | --- |
| HBV | M1 (Selected) | SARIMA (0,1,2)×(2,1,0)_12_ | 471.56 | 486.77 | 12 | 0 | 12 | 10.771 | 0.549 | 10.02 | 1.2020 |
| HBV | M2 | SARIMA (1,1,0)×(2,1,0)_12_ | 474.85 | 486.99 | 12 | 0 | 12 | 15.816 | 0.200 | 10.10 | 1.2170 |
| HBV | M3 | SARIMA (0,1,1)×(1,1,1)_12_ | 488.50 | 500.92 | 12 | 0 | 12 | 16.512 | 0.169 | 10.30 | 1.2280 |
| HBV | M4 | SARIMA (2,1,0)×(1,1,1)_12_ | 491.36 | 506.89 | 12 | 0 | 12 | 11.597 | 0.479 | 9.96 | 1.2130 |
| HBV | M5 | SARIMA (1,1,1)×(1,1,0)_12_ | 505.91 | 518.36 | 12 | 0 | 12 | 15.064 | 0.238 | 10.26 | 1.2380 |
| HCV | M1 (Selected) | SARIMA (1,1,1)×(0,0,1)_12_ | 77.95 | 90.96 | 24 | 3 | 21 | 31.162 | 0.074 | 15.85 | 0.2889 |
| HCV | M2 | SARIMA (1,1,1)×(1,0,0)_12_ | 76.66 | 89.66 | 24 | 3 | 21 | 35.374 | 0.028 | 15.79 | 0.2879 |
| HCV | M3 | SARIMA (0,1,2)×(1,0,0)_12_ | 77.78 | 90.79 | 24 | 3 | 21 | 38.715 | 0.010 | 15.85 | 0.2888 |
| HCV | M4 | SARIMA (1,1,2)×(1,0,0)_12_ | 78.07 | 94.33 | 24 | 4 | 20 | 35.351 | 0.018 | 15.77 | 0.2874 |
| HCV | M5 | SARIMA (2,1,1)×(1,0,0)_12_ | 78.11 | 94.37 | 24 | 4 | 20 | 34.845 | 0.020 | 15.75 | 0.2874 |

Note: SARIMA, seasonal autoregressive integrated moving average; AIC, Akaike information criterion; BIC, Bayesian information criterion; LB lag, maximum residual lag assessed in the Ljung–Box test; fitdf, fitted AR/MA parameters used for degrees-of-freedom adjustment; Test df, adjusted test degrees of freedom; MAPE, mean absolute percentage error; RMSE, root mean square error. Test df was calculated as LB lag minus fitdf. *P*<0.05 indicated evidence of remaining residual autocorrelation.

Supplementary Table S2. Sensitivity analysis of tourism–hepatitis associations after accounting for temporal trends in Hainan Province, 2005–2020

| Analysis | *n* | HBV Spearman *r* | 95% CI | *P* value | HCV Spearman *r* | 95% CI | *P* value |
| --- | --- | --- | --- | --- | --- | --- | --- |
| Raw annual level | 16 | 0.871 | (0.446, 0.955) | < 0.001 | 0.879 | (0.611, 0.991) | < 0.001 |
| Excluding 2020 | 15 | 0.857 | (0.536, 0.978) | < 0.001 | 0.896 | (0.563, 1.000) | < 0.001 |
| First-difference | 15 | 0.567 | (0.076, 0.874) | 0.028 | 0.07 | (-0.506, 0.693) | 0.804 |
| Linear detrended | 16 | 0.691 | (0.230, 0.914) | 0.003 | 0.421 | (-0.231, 0.964) | 0.105 |
